# Efficacy of Curcumin on Aortic Atherosclerosis: A Systematic Review and Meta-Analysis in Mouse Studies and Insights into Possible Mechanisms

**DOI:** 10.1155/2020/1520747

**Published:** 2020-01-07

**Authors:** Ke Lin, Huaijun Chen, Xiaojun Chen, Jinfu Qian, Shushi Huang, Weijian Huang

**Affiliations:** ^1^Department of Cardiology, The Key Lab of Cardiovascular Disease of Wenzhou, The First Affiliated Hospital of Wenzhou Medical University, Wenzhou, Zhejiang Province 325035, China; ^2^Department of Neurosurgery, Second Affiliated Hospital, School of Medicine, Zhejiang University, Hangzhou, Zhejiang Province 310000, China; ^3^Chemical Biology Research Center, School of Pharmaceutical Sciences, Wenzhou Medical University, Wenzhou, Zhejiang Province 325035, China

## Abstract

Since the first report in 2005, accumulating interests have been focused on the effect of curcumin in atherosclerosis with discrepancies. Therefore, we conducted a systematic review and meta-analysis to comprehensively estimate its effect against atherosclerosis. Literature search was performed on the database of PubMed, EMBASE, and Cochrane Library to identify relevant studies which estimated the effect of curcumin in atherosclerosis. Reporting effects on aortic lesion area was the primary outcome while effects on serum lipid profiles and circulating inflammatory markers were the secondary outcome. A total of 10 studies including 14 independent pairwise experiments were included in our analysis. We clarified that curcumin could significantly reduce aortic atherosclerotic lesion area (SMD = ‐0.89, 95% CI: -1.36 to -0.41, *P* = 0.0003), decrease serum lipid profiles (Tc, MD = ‐1.005, 95% CI: -1.885 to -0.124, *P* = 0.025; TG, MD = ‐0.045, 95% CI: -0.088 to -0.002, *P* = 0.042; LDL-c, MD = ‐0.523, 95% CI: -0.896 to -0.149, *P* = 0.006) as well as plasma inflammatory indicators (TNF-*α*, MD = ‐56.641, 95% CI: -86.848 to -26.433, *P* < 0.001; IL-1*β*, MD = ‐5.089, 95% CI: -8.559 to -1.619, *P* = 0.004). Dose-response meta-analysis predicted effective dosage of curcumin between 0 and 347 mg/kg BW per day, which was safe and nontoxic according to the existing publications. The underlying mechanisms were also discussed and might be associated with the modulation of lipid transport and inflammation in cells within artery walls as well as indirect modulations in other tissues. Clinical evidence from nonatherosclerosis populations revealed that curcumin would lower the lipid profiles and inflammatory responses as it has in a mouse model. However, standard preclinical animal trial designs are still needed; further studies focusing on the optimal dose of curcumin against atherosclerosis and RCTs directly in atherosclerosis patients are also warranted.

## 1. Introduction

Atherosclerosis is a disease mainly characterized by dysregulation of lipid metabolism, consequent chronic inflammation, and formation of atherosclerotic plaques in the arterial wall [1]. According to the current report, atherosclerosis and subsequent coronary heart diseases continue to be two of the major health concerns and affect more than 10% population worldwide [2]. Mechanisms driving the process of atherosclerotic lesion are complicated, including the activation of oxidative stress and low-grade inflammation [3, 4]. At present, therapeutic targeting drugs based on these mechanisms have been extensively implicated preclinically and clinically [5]. However, with plenty of efforts been made to improve the aortic injury of patients, little progress has been achieved.

Recently, emerging evidences have focused on dietary nutrition and natural compounds in improving atherosclerotic injury. Fiber, known as plant sterols and stanols, were found to be effective in decreasing LDL-c and improving the aortic atherosclerotic lesion area by affecting bile acid cycle [6]. Turmeric is a common herbal cuisine which has been used to treat various diseases since thousands of years ago in ancient China and India. As one of the main bioactive components of turmeric, curcumin [1,7-bis(4-hydroxy-3-methoxyphenyl)-1,6-heptadiene-3,5-dione] is now considered to be beneficial for health due to its activity of antiaging, anticancer, and anti-inflammation [7].

Intervention in the mouse model of atherosclerosis to evaluate the protective effect of curcumin on atherosclerosis was first developed by Olszanecki et al. in 2005 [8]. Subsequent studies were also carried out to confirm the protective function of curcumin on atherosclerosis [9–11]. However, another study found that curcumin could deteriorate the aortic lesion in APOE-deficient mice [12]. In addition, a randomized, double-blinded, and placebo-controlled trial designed by Mirzabeigi et al. indicated a little appreciable anti-inflammation effect of curcumin in patients with coronary heart disease [13]. The inconclusive effect of curcumin as well as the lacking appraisal of the quality of the reported data results in the necessity of evidence-based systematic review, which is still in the margin now.

Thus, we aimed to perform a systematic review and meta-analysis on curcumin and atherosclerosis or its associated diseases. However, few clinical studies [13, 14] have been completed, with focus more on serum lipid profiles or inflammation instead of a more direct and intuitionistic indicator of atherosclerosis, such as pulse wave velocity (PWV) and assessment of artery under angiography. Hence, we performed a systematic review and meta-analysis to investigate the effect of curcumin for atherosclerosis in mouse models and further discussed the potential cellular and molecular mechanisms.

## 2. Methods

### 2.1. Study Selection

The systematic review was carried out step by step in accordance with SYRCLE's systematic review protocol format for animal intervention studies (https://www.syrcle.nl) [15].

Specifically, a systematic literature search was conducted in PubMed, EMBASE, and Cochrane Library databases from the earliest available date to April 2019, using the following keywords and subject terms: (atherosclerosis OR atherogenesis) AND (mice OR mouse) AND (curcumin OR curcuma OR turmeric yellow). The reference lists of included studies were also searched manually to identify additional relevant studies.

Two individual reviewers (Ke Lin and Huaijun Chen) screened the titles and abstracts to pick out the potential studies. Duplicate studies were removed after screening the title and abstract of each article. Full-text manuscripts were subsequently reviewed for available data according to the eligibility criteria. Disagreements were resolved by consensus with a third author (Jinfu Qian).

### 2.2. Eligibility Criteria

#### 2.2.1. Types of Studies

All studies were restricted to original articles, and conference proceedings, commentaries, and reviews were excluded. Clinical cases or trails, solely *in vitro* studies or animal studies which were not related to the topic, were also excluded.

#### 2.2.2. Types of Participants

Small animals other than mice were ruled out in this systematic review. Laboratory mice of any age and gender were all included. No specific restriction was imposed to the model of atherosclerosis.

#### 2.2.3. Types of Interventions

A pairwise comparison between curcumin-treated atherosclerotic mice and placebo-treated atherosclerotic mice must be designed in the selected studies. Any means of curcumin administration, including gavage, intravenous injection, or dietary supplements, were included. All types of curcumin were included except for curcumin mixture, for example, the turmeric extracts (including curcuminoids) and the herbal mixture (Artemisia iwayomogi Kitamura and Curcuma longa Linne). The dosage and time of curcumin intervention were not limited. If the study contained more than one dosage of curcumin, then each dosage of curcumin was considered a separate experiment.

#### 2.2.4. Types of Outcome Measures

An aortic lesion area was defined as the main visualized indicator for atherosclerosis. Hematoxylin-eosin staining (HE) or Oil Red O (ORO) staining of the whole aorta or the presentative part of aorta, such as aortic sinus, was acceptable while studies without the available data of atherosclerotic lesion area were ticked out. Biochemical analysis of atherosclerosis was compromised by serum inflammation levels and lipid levels. Quantitative detection of serum total cholesterol (Tc), triglyceride (TG), low-density lipoprotein (LDL-c), high-density lipoprotein (HDL), tumor necrosis factor-*α* (TNF-*α*), interleukin-1*β* (IL-1*β*), and interleukin-6 (IL-6) represented the lipid level and inflammation level.

The primary outcome of this meta-analysis was the curcumin effect on the aortic atherosclerotic lesion area, and the secondary outcome was the change of serum biochemical indicators after curcumin administration.

### 2.3. Data Extraction

Two individual researchers (Ke Lin and Shushi Huang) extracted the detail data of all included studies. The detailed data contained the following: first author's name and publication date, mouse age, gender, gene modification and diet, administration dosage, route and duration of curcumin, sample numbers, assessing location of atherosclerosis, aortic lesion staining methods, proportion of aortic staining positive area, and measured serums TNF-*α*, LDL-c, and HDL of the placebo-controlled and curcumin-treated groups. The data was collected by the mean value, standard deviation (SD), and the number of animals per group. If the data was shown as the mean ± SE, then we would transform the raw data into the mean value and SD, according to the principles of statistics.

### 2.4. Quality and Bias Assessment in Individual Studies

Two individual authors (Ke Lin and Huaijun Chen) assessed the risk of bias of all included studies by using the SYRCLE's risk of bias tool, as reported by Chen et al. [16]. In detail, random sequence generation, baseline characteristics, allocation concealment, random housing, blinded interventions, random outcome assessment, blinding of outcome assessment, incomplete outcome data, selective reporting, and other bias were estimated. For each of the item, the answer yes indicated low risk of bias, no indicated high risk of bias, and “unclear” indicated that the risk of bias was not clear. Disagreements of the quality assessment were resolved by discussion with a third author (Xiaojun Chen).

### 2.5. Data Synthesis and Statistical Analyses

As different dosages of curcumin treated in a single study were defined as several independent experiments, we subsequently divided the animal numbers of the placebo-controlled group by the number of dosages to avoid a man-made expansion in sample size. Particularly, in two studies [17, 18], researchers used the same atherosclerosis mouse model group as the control group in comparison with the other three dosages of the curcumin group, and thus, the number of control animals was divided into three groups. In addition, different units of curcumin dosage were used in the selected studies due to different administration routes and needed standardizing for further subgroup analysis. As is mentioned by Sharma et al. [19], a 0.2% dietary curcumin approximately equaled to 300 mg/kg BW in oral gavage for mice, and only 60% of oral curcumin could be absorbed and detected in serum. In this manner, we transformed the dosages of dietary curcumin and injected curcumin into a standard dosage of oral curcumin.

Review Manager 5.3 software and Stata 15.0 software were used in our meta-analysis. We performed a separate pairwise meta-analysis in the outcome of the aortic lesion area and biochemical indicators, and the random effects model (*I*^2^ > 50%) or the fixed effects model (*I*^2^ ≤ 50%) was used during the process of continuous data synthesis. The standardized mean difference (SMD) was applied to calculate the aortic lesion area due to different staining methods and different assessing location among studies. And the mean difference (MD) was employed to measure biochemical indicators. Data were expressed as the SMD or MD with 95% confidence intervals (95% CI). The effect of curcumin on the aortic atherosclerotic lesion area was shown in the forest plot, and its effects on serum biochemical indicators were shown in tables.

Heterogeneity was assessed by Cochrane's *I*^2^. When *I*^2^ was above 50%, then the result was considered to have a high level of heterogeneity [20]. To explore potential causes of high heterogeneity, standardized curcumin dosage subgroup analyses were performed when each of the subgroup contained more than three studies. A further meta-regression was also proceeded. For continuous variables and binary variables, metaregression was conducted directly in Stata. For multicategorical variables, a dummy variable was first set and metaregression was subsequently performed. When the *P* value was less than 0.05, then the variable was considered a source of heterogeneity. Moreover, we applied sensitivity analysis to evaluate whether the findings were robust enough to draw the conclusion. Publication bias was detected using the funnel plot and Egger's test (*P* < 0.05 was considered to indicate publication bias).

The relationship between the standard dosage of curcumin and the ratio of means (RR = means_experimental_/means_control_) was modeled using a nonlinear quadratic regression, as descried by Liu et al. [21]. In detail, the regression curve passed the following equation: lnRR = *β*1*x* + *β*2*x*^2^ + *ε*, and the generalized least squares method (GLS) was used to calculate each of the parameters while *R*^2^ represents the degree of fitting.

## 3. Results

### 3.1. Selection of Studies

**Figure 1:**
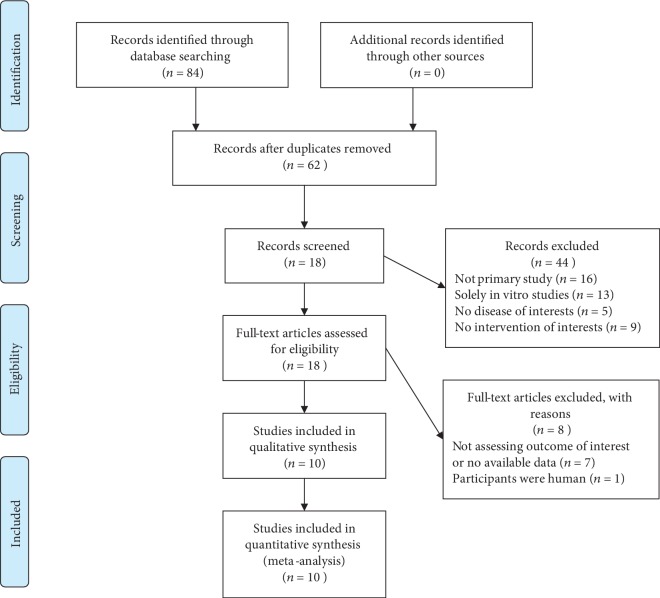
Flowchart of the literature search and selection.

The detailed flow diagram of literature identification and selection process is shown in Figure 1. Following the complete searching strategy, a total of 41 records from PubMed, 43 records from EMBASE, and 0 records from Cochrane Library were imported into Endnote X9, and 62 studies were identified after the duplication removal. We screened for titles and abstracts and picked out 18 studies for further full-text manuscript selection. Among these, 8 studies [11, 22–27] were excluded due to no available outcome data that met our inclusion criteria, and one study [28] was excluded because of the improper participants (not mice). Thus, we included 10 eligible studies [8, 9, 12, 17, 18, 29–33] in this systematic review and meta-analysis. Two of the studies [17, 18] contained multiple dosages of curcumin and were then considered a total of six independent experiments as we have mentioned in the eligibility criteria. At last, a total number of 14 comparative experiments between the placebo-controlled group and curcumin group and 235 mice were involved in this meta-analysis.

### 3.2. Characteristics of the Studies Included in the Meta-Analysis

Detailed characteristics of all included studies are summarized in Table 1.

Among these 10 studies, three types of mice were used, including APOE^−/−^, LDLR^−/−^, and APOE^−/−^ LDLR^−/−^ mice, in which the APOE^−/−^ mice were the most common one (8/10). Only one study [8] used female mice as the participants while 8/10 used male mice. All the mice were treated with high-fat diet to induce atherosclerosis, except for Sawada et al. [12], in which the model of atherosclerosis was spontaneously developed in APOE^−/−^ mice fed a normal rodent chow diet.

Curcumin varied from sources, dosages, and routines among these studies. Seven of studies purchased the curcumin from Sigma-Aldrich, and 2 studies used the curcumin from Wako Pure Chemical Industries or Caymen Chemical while the other one did not mention the source of curcumin. Curcumin used in each study was isolated from turmeric as the companies claimed, with a purity of more than 90%. The curcumin was administrated in different ways, such as in oral gavage, supplemented in diet, or by intravenous injection. Most studies (8/10) chose the time of more than 10 weeks as the administration time. The dosage was the main variable factor among studies within a range from 3.3 mg/kg per day (standardized in BW) to 750 mg/kg BW per day. Comprehensively, we divided all the curcumin-treated groups into three concentrations mostly in consideration of the standardized dosages per day. In detail, a dosage of more than 200 mg/kg BW per day was considered a high curcumin-treated groups, and a dosage of less than 100 mg/kg BW per day was defined as a low curcumin-treated groups, while the dosage in between was considered medium.

All studies employed a quantitative method to assess the aortic atherosclerotic lesion area, in which 8/10 used Oil Red O to stain the aorta while the others applied hematoxylin-eosin or Sudan III. All the included studies detected at least one of the plasma lipid profiles or inflammatory makers, except for Sawada et al. [12].

### 3.3. Risk of Bias and Quality of Included Studies

**Figure 2:**
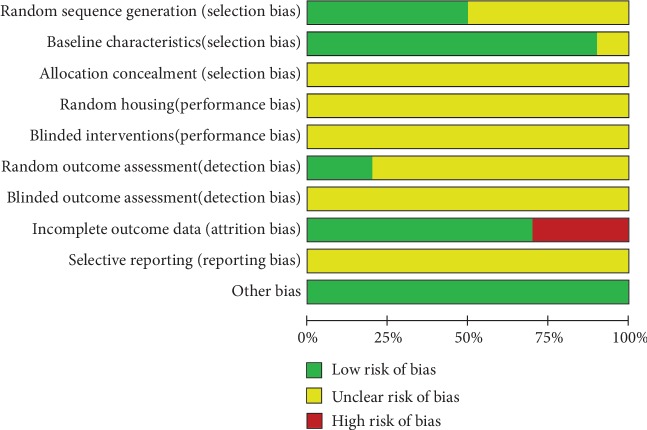
Risk of bias and quality assessment.

According to the criteria of SYRCLE's risk of bias tool [16], the quality of included studies was graded. Five studies (5/10) were graded as low in sequence generation as they declared that the animals were randomly divided into different groups. However, in the assessment of allocation concealment and random housing, all studies manifested unclear risks, since they did not clearly describe whether the allocation was concealed or whether the animals were randomly housed. Nine studies (9/10) had low risk of selection bias, as they described all animal characteristics and make sure that mice were similar in the baseline. All studies (10) did not mention the blinded methods either in drug-intervention or in outcome assessment. Two studies (2/10) declared that animals were randomly selected for different outcome assessments, thus owning low risk of detection bias. However, all of studies did not state whether the studies were free of selective outcome reporting. Three studies (30%) achieved a high rating by providing outcome data of part of animals while the other 7 studies were graded as low. On account of the risk of other biases, all studies (10) were rated as low. The overall results are shown in Figure 2.

### 3.4. Protective Effect against Aortic Atherosclerosis of Curcumin

**Figure 3:**
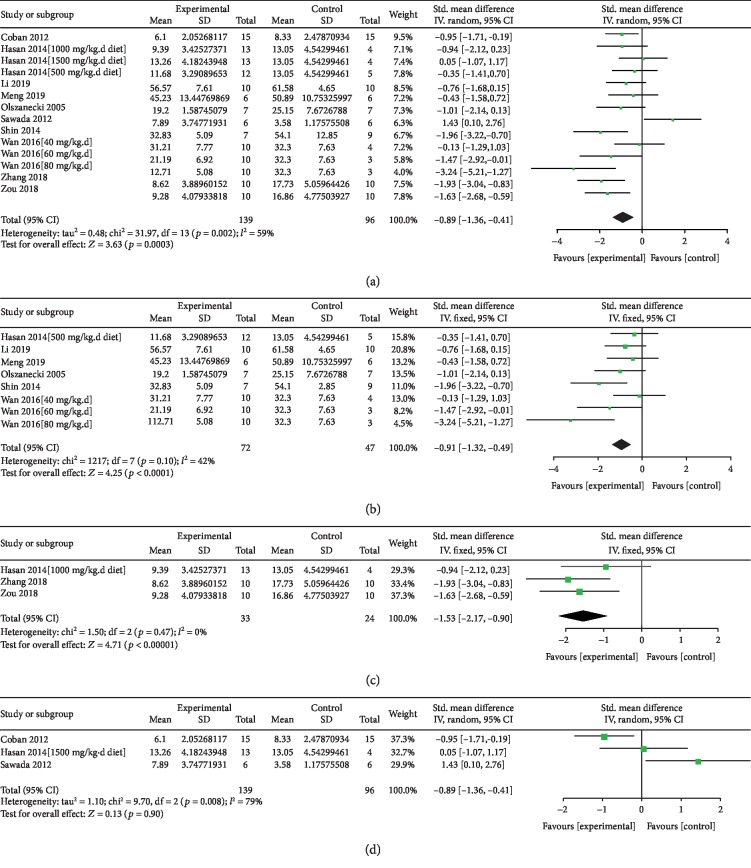
Forest plot of curcumin on aortic lesion area. (a) Overall effect of curcumin. (b) Subgroup analysis of low standardized dosage curcumin. (c) Subgroup analysis of medium standardized dosage curcumin. (d) Subgroup analysis of high standardized dosage curcumin. Low standardized dosage, less than 100 mg/kg per day BW; medium standardized dosage, between l00 mg/kg per day BW and 200 mg/kg per day BW; high standardized dosage, more than 200 mg/kg per day BW; SD: standard deviation; Std.: standard; IV: inverse variance; CI: confidence interval. *P* < 0.05 was considered to be statistically different.

Atherosclerotic lesion data from 14 comparisons were synthesized as shown in Figure 3. An overall effect of curcumin was to significantly decrease the atherosclerotic lesion area (SMD = ‐0.89, 95% CI -1.36 to -0.41, *P* = 0.0003, Figure 3(a)). However, the heterogeneity was high with *I*^2^ of 59%. Thus, we carried out a subgroup analysis based on the concentration of standardized dosage. In both the low-dosage group and medium-dosage group, the effect of curcumin was the same to be protective (Figures 3(b) and 3(c)). And the heterogeneity between studies in these subgroups was acceptable (*I*^2^ = 42%, 0%). However, in the high-dosage group, there might be no protective effect of curcumin (*P* = 0.9, Figure 3(d)). In this manner, we found that the main heterogeneity between studies was from the different dosages of curcumin. To further explore the potential factors contributing to the heterogeneity, we conducted metaregression analysis. As shown in Table 2, a total of 8 potential variables were brought into the analysis, and none of them resulted in the high heterogeneity with *P* > 0.05.

**Figure 4:**
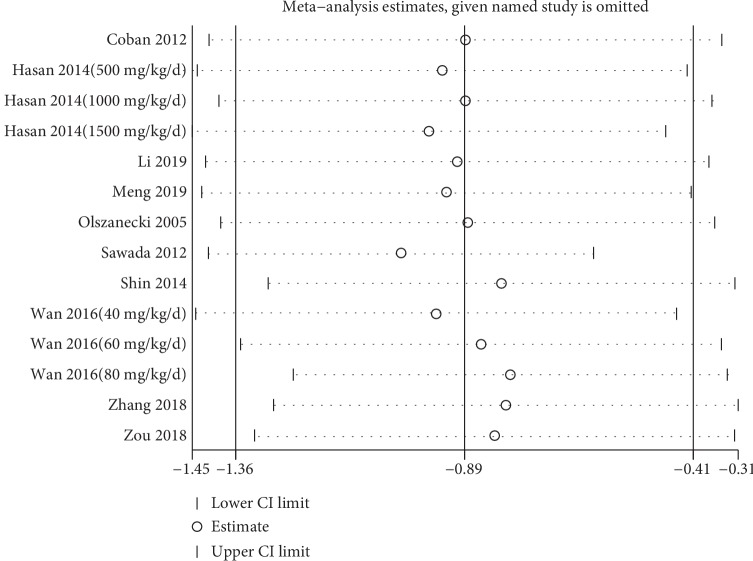
Sensitivity analysis of curcumin effect on aortic atherosclerotic lesion area. Meta-analysis estimated when each of the study was omitted. CI: confidence interval.

**Figure 5:**
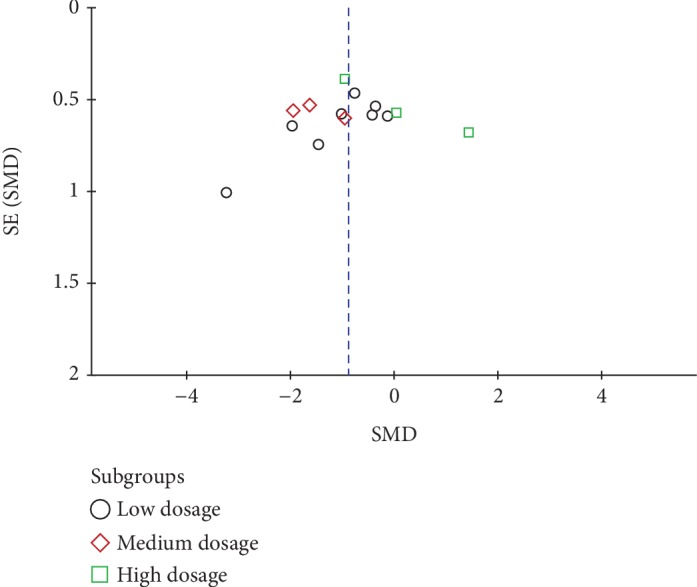
Funnel plot of the included studies. SMD: standard mean difference.

In order to test the reliability of our conclusion, we carried out the sensitivity analysis. With each of the study being omitted, no significant change was found in the estimating outcomes, which indicated the robustness of our study (Figure 4). Moreover, a funnel plot overseeing the publication bias was conducted. The symmetry of the plot suggested that no publication bias existed (Figure 5). Additionally, we proceeded Egger's test, and the result was consistent with the funnel plot with *P* = 0.587.

**Figure 6:**
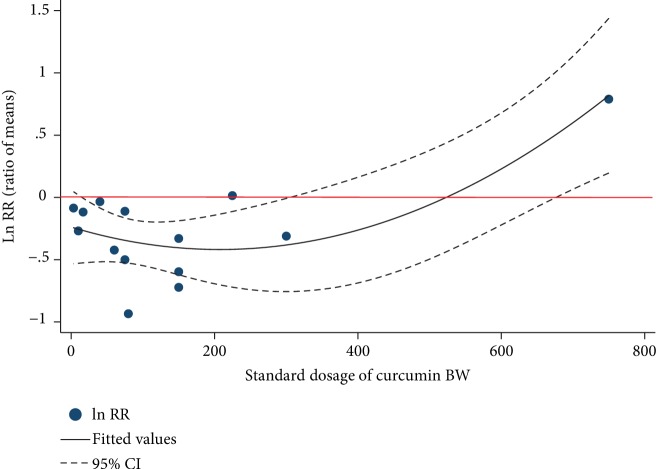
Nonlinear dose-response relationship between Napierian logarithm of RR and the intake of curcumin. The red line represents an invalid effect. The area between the dash lines represents the 95% CI. Dots represent each of the studies. RR: ratio of means, equal to means_experimental_/means_control_.

Since the dose of curcumin was the main source of heterogeneity and the dose of curcumin might influence the effect as many researchers have reported [17, 18], we conducted a dose-response meta-analysis. Firstly, a univariate metaregression analysis was performed to clarify the relationship between the SMD of the aortic lesion area and overall dosage of curcumin included in our analyses. As shown in [Supplementary-material supplementary-material-1], the effect seemed to be positively correlated with the standard dosage of curcumin BW, which was inconsistent with the previous reports [17, 18]. Thus, we supposed that a nonlinear association might be appropriate for the relationship of dosage and protective effects of curcumin. According to the method of quadratic regression of meta-analysis [21], RR, defined as the ratio of means (means_experimental_/means_control_), was recalculated. As shown in Figure 6, the protective effect of curcumin against aortic atherosclerotic lesion was strengthened and then weakened or even reversed as the dosage of curcumin increased. In our model, 207 mg/kg BW was the optimized dose of curcumin with the best predicted protective effect while a dosage of more than 347 mg/kg BW would further aggravate the aortic lesion. The predicted relationship could be expressed as lnRR = 4.20∗10^−6^*x*^2^ − 0.0017*x* + 0.1351, *R*^2^ = 0.5944.

### 3.5. Antagonism of Inflammation and Hyperlipidemia Participates in Curcumin Promoting Atherosclerotic Lesion

Since high lipid profiles in the serum and continuous chronic inflammation in the vessel were the major pathological contribution to aortic atherosclerotic lesion, we collected these data from all viable included studies and carried out the meta-analysis. As shown in Table 2, curcumin could significantly lower the concentration of Tc, TG, and LDL-c (Tc, MD = ‐1.005, 95% CI: -1.885 to -0.124, *P* = 0.025; TG, MD = ‐0.045, 95% CI: -0.088 to -0.002, *P* = 0.042; LDL-c, MD = ‐0.523, 95% CI: -0.896 to -0.149, *P* = 0.006), while there might be no improvement on HDL (MD = ‐0.087, 95% CI: -0.235 to 0.060, *P* = 0.245). Further sensitivity analysis ([Supplementary-material supplementary-material-1], Table 3), and Egger's test revealed that the conclusion was robust with no publication bias (Tc, *P* = 0.226; TG, *P* = 0.063; LDL-c, *P* = 0.975; HDL, *P* = 0.081). For plasma inflammatory responses, curcumin was found to be efficient to decrease serum TNF-*α* and IL-1*β*, while the effect on IL-6 was useless with *P* = 0.108 (MD = ‐10.687, 95% CI: -23.705 to 2.331). In addition, sensitivity analysis and Egger's test showed that the conclusion of IL-6 and IL-1*β* was reliable (Figures [Supplementary-material supplementary-material-1], Table 3). However, in the outcome of plasma TNF-*α*, when including all 8 independent pairwise experiments, the outcome was not robust because when omitting the data from Hasan et al. [17], the estimating MD became out of the confidence interval ([Supplementary-material supplementary-material-1]). Therefore, 2 pairwise experiments from Hasan et al. [17] were omitted. Although Egger's test found that the publication bias existed in the result of plasma TNF-*α* with *P* = 0.036, subsequent sensitivity analysis revealed that the outcome was robust ([Supplementary-material supplementary-material-1]). In this part, we found that curcumin could influence part of the serum lipid profiles and inflammatory responses.

## 4. Discussion

### 4.1. Summary of the Current Findings

To our knowledge, this is the first systematic review and meta-analysis to estimate the therapeutic effect of curcumin on atherosclerosis in a mouse model. Upon 10 researches consisting of 14 pairwise experiments and 235 mice, we found that curcumin could significantly decrease the aortic atherosclerotic lesion area as well as the serum lipid profiles (Tc, TG, and LDL-c) and inflammatory levels (TNF-*α* and IL-1*β*). Notably, not all lipid profiles and inflammatory markers (HDL and IL-6) would be affected in our findings, which indicated that there might be selective pathways through which curcumin decreased the plasma lipid level and chronic inflammation. On the other hand, it might also be because of the fact that few studies were included in our analyses. Although researchers have already used curcumin as a positive drug to treat atherosclerosis [34], it still needs comprehensive consideration as we found that there was a dose-response relationship between curcumin and its protective effect on atherosclerosis. Based on our results, the effect of curcumin on decreasing aortic lesion area became stronger in low and medium dosages (predicted to be less than 207 mg/kg BW per day) and would be weaker when the dose was more than 207 mg/kg BW per day, even to aggravate the disease when the dose reached 347 mg/kg BW per day. This interesting finding was consistent with some of the researches [17, 18] compromising more than one dose of curcumin groups. And we supposed that this was why some researchers [12] found that curcumin was harmful for treating atherosclerosis. In summary, we demonstrated that curcumin could function as a protective role against atherosclerosis in a certain range of dosage (0 to 347 mg/kg BW per day, as predicted).

### 4.2. Heterogeneity

Differences in experimental designs and assessing methods often resulted in the heterogeneity in preclinical studies [35]. In this systematic review and meta-analysis, we found out the potential evidence of heterogeneity among included studies by performing subgroup analysis and subsequent metaregression analysis. For the outcome of the aortic lesion area, subgroups were divided based on the standardized dosage of curcumin as mentioned in the Methods section. As expected, heterogeneity was remarkably reduced in subgroups of low and medium dosages, while it remained high in the subgroup of high dosage, which indicated that the dosage of curcumin was the main source of heterogeneity. Moreover, we explored whether other factors varied in different studies contributed to the heterogeneity. Ages and gender of mice were confirmed to significantly influence the development of atherosclerosis [36]. A different assessing location of the aortic lesion was also believed to be one of the potential sources of heterogeneity as atherosclerotic plaques preferred to appear in the aortic root [37]. Other different variables among studies included publication year, study length, source of curcumin, staining methods, and route of curcumin. A complete meta-regression analysis was performed, and none of these variables significantly contributed to the heterogeneity.

### 4.3. Safety and Toxicity of Curcumin Administration

As we predicted, an effective concentration of curcumin was ranged from 347 mg/kg BW per day, especially 207 mg/kg BW per day. It was important to see whether it was safe enough. Lots of animal studies have reported no significant toxicity of curcumin. Wahlstrom and Blennow reported that oral doses up to 5 g/kg of curcumin showed no significant toxicity in Sprague–Dawley rats [38]. Similar findings were validated in dogs or donkeys with the concentration of 3.5 g/kg BW for a continuous curcumin administration of 3 months [39]. In mice, 0.2% dietary curcumin (approximately 300 mg/kg BW) exhibited no toxicity [40]. Furthermore, curcumin at the single dose of 5000 mg/kg body weight that was given orally to Swiss albino mice did not show any toxic effects during 14 days with no pathological effects observed and no deaths occurred [41]. In addition, 500 mg/kg BW, 1000 mg/kg BW, and 2000 mg/kg BW of oral curcumin showed no significant genotoxicity in mice [42].

Hepatotoxicity, decreases in weight, and reduction of hemocytes were the main side effects observed in animals as summarized by Soleimani et al. [43]. However, all these mild adverse events were observed in administration with turmeric extracts, which were the mixture of curcuminoids and other components. In human, common side effects such as flatulence [44], redness of tongue [45], and changes in liver enzymes and biochemical parameters of blood [46] were observed when treated with purified nanocurcumin. Hence, we found that the liver might be mainly vulnerable in curcumin treatment, and we supposed that this was associated with its pharmacokinetics as Ireson et al. [47] demonstrated that curcumin was firstly reduced to hexahydrocurcumin in the liver. Furthermore, many of its hypolipidemic effects were also functioned in the liver [25]. Thus, it would be a great burden for the liver if the concentration was too high.

Taken all these evidences together, it would be safe with only few acceptable side effects at least in mice when oral curcumin reached our predicted concentrations.

### 4.4. Investigations into Clinical Trials

Despite the fact that few studies were conducted to assess the effect of curcumin on atherosclerosis, numerous clinical trials focusing on curcumin also estimated serum indicators associated with atherosclerosis. Although some studies demonstrated that curcumin has a little appreciable effect on atherosclerosis [13, 48], more trials reported an atheroprotective effect of curcumin [49–52]. Furthermore, researchers have found that curcuminoids (curcumin > 70%) could decrease the atherogenic risk. In a 6-month, randomized, double-blinded, and placebo-controlled clinical trial, patients with diabetes were treated with curcuminoids or placebo. The results showed that curcumin intervention significantly reduced pulse wave velocity, TG, and other parameters and thus lowered the atherogenic risks [53]. In another study, Panahi et al. found that curcuminoid supplementation can reduce serum levels of atherogenic lipid indices including non-HDL and lipoprotein A and in turn contributed to the reduction of atherogenic risk in dyslipidemia patients with diabetes [54]. Besides, Panahi et al. also demonstrated that curcumin supplementation could decrease the leptin/adiponectin (a measure of atherosclerosis) [55]. However, deficiency of direct investigations of pure curcumin in atherosclerosis populations limited the conclusion.

### 4.5. Possible Cellular and Molecular Mechanisms of Curcumin against Atherosclerosis

It was widely accepted that macrophages (M_*φ*_), endothelial cells (ECs), and vascular smooth muscle cells (VSMCs) were the predominant cells responsible for the pathogenesis of atherosclerosis [56]. Triggered by dyslipidemia and injury of endothelial cells, LDL-c, and oxidative LDL-c (ox-LDL) were accumulated and stimulated the secretion of several critical cytokines. For example, vascular cell adhesion molecular-1 (VCAM-1), intercellular adhesion molecular-1 (ICAM-1), and monocyte chemotactic protein-1 (MCP-1), secreted by ECs and VSMCs, were believed to be essential in the accumulation of macrophages which would further activate the inflammation [57]. Mediated by scavenger receptor (SR-A), CD36, toll-like receptors (TLRs) [58, 59], macrophages could engulf the lipid and transformed into foam cells. The apoptosis of foam cells released the lipid and intensified the inflammation and oxidative stress in the susceptible sites of the artery wall [60]. Thus, dysfunction of ECs, overproliferation of VSMCs, accumulation of LDL, and macrophages resulted in the inflammation and the formation of prototypical atherosclerotic plaques.

As an atheroprotective compound, curcumin functioned mainly by lowering the lipid profiles and inhibiting inflammation in all these cells aforementioned. In our analysis, we observed a significant decrease of Tc, TG, and LDL in a mouse model of atherosclerosis (*P* = 0.025, 0.042, and 0.006, respectively), which was consistent with the evidence of clinical trials [61]. Enterohepatic circulation of cholesterol and the function of gut microbiota were confirmed to be responsible for this effect of curcumin, as found by Ghosh et al. [23]. Macrophage was one of the main targets of curcumin. Zhao et al. [26] revealed that curcumin could affect the transport of cholesterol by upregulating the expression of ATP-binding cassette transporter A1 (ABCA1) and inhibiting SR-A, CD36, and abrogated the formation of foam cells. Inhibition of TLR4 by curcumin would further reduce inflammation in APOE^−/−^ mice [31]. In addition, curcumin could decrease the expression of adiponectin protein 2 (AP2), which in turn abolished the activation of endoplasmic reticulum (ER) stress and downstream NF-*κ*B, MAPK, therapy depressing the release of inflammatory cytokines (including TNF-*α* and IL-1*β*) as well [62]. In VSMCs, emerging evidences have demonstrated that curcumin could inhibit migration and proliferation. A dose-dependent inhibition (curcumin 5-20 *μ*M) of migration was observed in primary VSMCs, isolated from the aorta of Sprague–Dawley rats [63]. In another study, Li et al. [64] found that curcumin could significantly inhibit the proliferation of rat VSMCs by elevating peroxisome proliferator-activated receptor-*γ* (PPAR-*γ*) and reducing oxidative stress. Moreover, insulin-like growth factor 1 receptor (IGF-1R) was detected to be reduced in human plaque intimal VSMCs while increasing expression of IGF-1R-rescued plaque VSMCs from oxidative stress-induced apoptosis [65], which was strongly associated with the plaque stability. Curcumin could activate Forkhead Box O (FOXO) transcription and increase the expression of IGF-1R and in turn stabilize the plaque [62]. As for endothelial cells, curcumin was confirmed to be efficient for the decrease of ICAM-1 through a HO-1-independent pathway [66]. Another *in vivo* study conducted by Min et al. also validated that curcumin could reduce the mRNA and protein expression of ICAM-1, VCAM-1, MCP-1, and P-selectin [67]. In this manner, adhesion of monocytes with ECs in the susceptible sites of the artery wall was hampered by curcumin.

In addition, curcumin could also function as an antiatherogenic compound in other tissues. Ghosh et al. [23, 24] reported that curcumin (100 mg/kg per day BW, 6 weeks) could improve the function of the intestinal barrier preventing glucose intolerance, as well as decreasing circulatory intestine-derived lipopolysaccharide (LPS), thus ameliorating aortic lesion in LDLR^−/−^ mice. Another study [11] performed on LDLR^−/−^ mice also demonstrated that curcumin could inhibit hepatic expression of HMG-CoA (3-hydroxy-3-methyl-glutaryl-co-enzyme A) reductase and finally improved the symptoms of aortic atherosclerosis. Besides, Zingg et al. [25] found that curcumin could restore the suppression of cyclic adenosine monophosphate (cAMP) induced by HFD in the liver and adipose tissue (but not in the brain, skeletal muscle, spleen, and kidney), thus activating the cAMP/PKA/CREB pathway as well as strengthening lipolysis and fatty acid beta-oxidation in the tissues and contributing to its hypolipidemic effects and improvement of aortic atherosclerosis.

**Figure 7:**
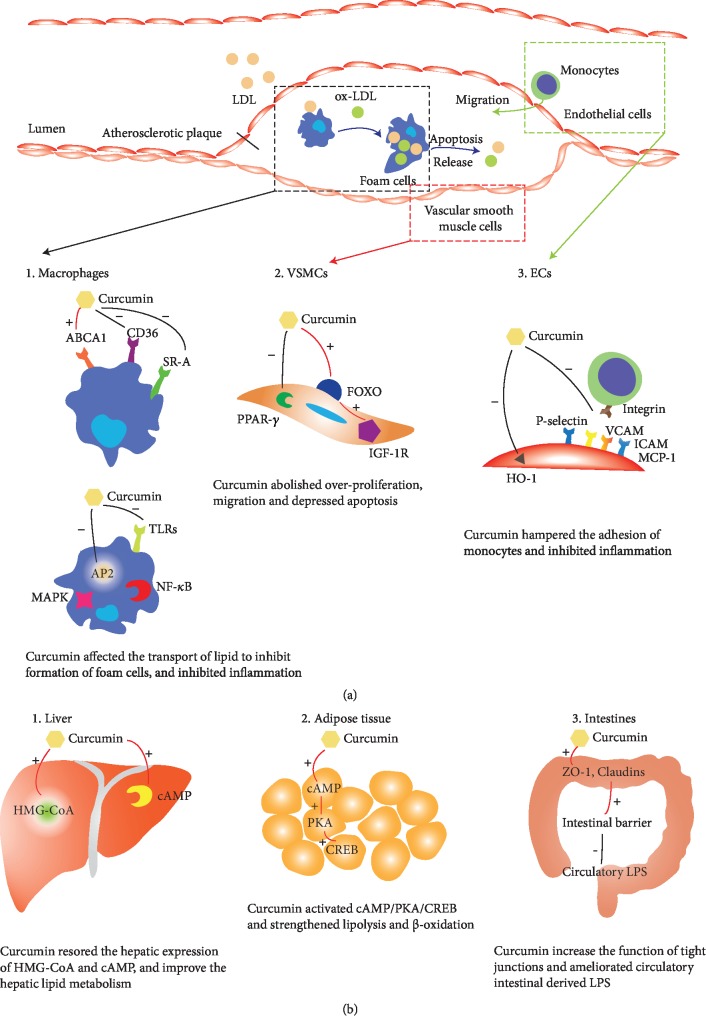
Diagrammatic sketch of the possible molecular mechanisms driving the protective effect of curcumin against aortic atherosclerosis. (a) Effects of curcumin in susceptible artery wall. (b) Effects of curcumin in other tissues.

Taken together, curcumin could affect the function of VSMCs, ECs, and M_*φ*_ in the susceptible artery walls as well as hepatocytes and other types of cells in other tissues, by several signaling pathways, and in turn decrease lipid profiles and inflammation responses in the susceptible area in the vessels, thus improving aortic lesion. The diagrammatic sketch of the possible mechanisms mentioned above was summarized and is shown in Figure 7.

### 4.6. Limitations

Although this analysis was conducted seriously according to the handbook of systematic review for animal intervention studies [15], there were still limitations. First, even though we searched exhaustively in the database of PubMed, EMBASE, and Cochrane library, some of grey literatures including conference proceedings or unpublished studies or published studies lacking of the data of aortic lesion area were still out of our inclusion; thus, we could not be sure all relevant studies were found. In addition, lacking data of antioxidant indicators among included studies restricted our analysis now that oxidative stress might also play a central role in atherosclerosis lesion. Thirdly, the poor quality of included researches in our analysis might also be a source of heterogeneity and influence our conclusion. Since many items in the section of risk and quality assessment were unclear, further efforts were needed to standardize the design and implementation of the animal intervention experiments. More importantly, the mouse model of atherosclerosis was still different from atherosclerosis in human beings in some aspects. For instance, the atherosclerotic plaque of humans tended to occur in the coronary artery while it would gather in the aortic root in mice [37]. Besides, intervention of curcumin in mice was started before the disease onset while the starting time of therapy in human beings could hardly reach. Notably, clinical investigation in nonatherosclerosis patients rather than the direct one was another limitation. All these limitations would somewhat restrict the implication of curcumin.

## 5. Conclusion

Curcumin treatment was shown to be effective for decreasing the aortic atherosclerotic lesion area as well as for lowering lipid profiles and inflammation in the mouse model of atherosclerosis, despite the poor quality of the included studies. Comprehensively, the mechanisms driving the protective process of curcumin might be related to the modulation of macrophages, VSMCs, and ECs around the atherosclerotic plaques. It is worth noting that the protective effect was remarkably associated with the dose of curcumin, and a medium dosage of curcumin (between 100 and 200 mg/kg BW per day) was confirmed to be better, while the optimal dosage of curcumin was predicted to be 207 mg/kg BW per day. But it needs further optimal dose-setting study to confirm the result. Additionally, poor quality of the studies in this analysis also called for standardized designing guidelines in preclinical studies of atherosclerosis. Moreover, high-quality clinical evidence directly based on atherosclerosis population RCTs was needed before dietary supplementation of curcumin become a reliable therapeutic schedule.

## Figures and Tables

**Table 1:** Characteristics of all included studies.

Studies	Gene modification	Age	Gender	Diet	Route	Source Purity	Study length	Dosage	Standardized dosage (BW)^∗^	Quantification of atherosclerotic lesion	Detection of biochemical indicators	Included groups and sample sizes
Coban 2012	APOE^−/−^	8 w	Male	HFD	Chow	Sigma ≥98%	16 w	2000 mg/kg/d dietary	300 mg/kg	Aortic sinus stained with Oil Red O	Lipid markers (Tc, TG)	Model control, *n* = 15 Curcumin, *n* = 15
Hasan 2014	LDLR^−/−^	8 w	Male	HFD	Chow	Sigma ≥98%	16 w	500 mg/kg/d dietary	75 mg/kg	Whole aorta stained with Oil Red O	Lipid markers (Tc, TG, HDL) Inflammation markers (TNF-*α*, IL-6, IL-1*β*)	Model control, *n* = 24 Curcumin low, *n* = 24 Curcumin medium, *n* = 24 Curcumin high, *n* = 24
								1000 mg/kg/d dietary	150 mg/kg	Whole aorta stained with Oil Red O		
								1500 mg/kg/d dietary	225 mg/kg	Whole aorta stained with Oil Red O		
Li 2019	APOE^−/−^	8 w	Male	HFD	i.v.	Sigma ≥98%	30 d	2 mg/kg/3d BW	3.3 mg/kg	Thoracic aorta stained with Oil Red O	Lipid markers (Tc, TG, LDL)	Model control, n = 10 Curcumin-liposomes, n = 10
Meng 2019	APOE^−/−^	8 w	Male	HFD	i.v.	/	6w	10 mg/kg/d BW	16.7 mg/kg	Whole aorta stained with Oil Red O	Lipid markers (Tc, TG, HDL, LDL) Inflammation markers (TNF-*α*, IL-6)	Model control, *n* = 6 Curcumin, *n* = 6
Olszanecki 2005	APOE^−/−^ LDLR^−/−^	8 w	Female	HFD	Chow	Cayman Chemical ≥90%	16 w	0.3 mg/d/mouse BW	10 mg/kg	Whole aorta stained with Oil Red O	Lipid markers (Tc, TG)	Model control, *n* = 10 Curcumin, *n* = 10
Sawada 2012	APOE^−/−^	5 w	Male	NCD	Chow	Wako Pure Chemical Industries ≥90%	15 w	5000 mg/kg/d dietary	750 mg/kg	Whole aorta stained with Sudan III	/	Model control, *n* = 12 Curcumin, *n* = 12
Shin 2014	APOE^−/−^	7 w	Male	HFD	Chow	Sigma ≥98%	10 w	50 mg/kg/d dietary	75 mg/kg	Aortic arch stained with Oil Red O	Lipid markers (Tc, TG, HDL, LDL) Inflammation markers (TNF-*α*, IL-6)	Model control, *n* = 9 Curcumin, *n* = 7
Wan 2016	APOE^−/−^	6 w	Male	HFD	i.g.	Sigma ≥98%	12 w	40 mg/kg/d BW	40 mg/kg	Aortic arch stained with HE	Lipid markers (Tc, TG, HDL, LDL) Inflammation markers (TNF-*α*, IL-6)	Model control, *n* = 10 Curcumin low, *n* = 10 Curcumin medium, *n* = 10 Curcumin high, *n* = 10
								60 mg/kg/d BW	60 mg/kg	Aortic arch stained with HE		
								80 mg/kg/d BW	80 mg/kg	Aortic arch stained with HE		
Zhang 2018	APOE^−/−^	9 w	Male	HFD	Chow	Sigma ≥98%	16 w	1000 mg/kg/d dietary	150 mg/kg	Aortic sinus stained with Oil Red O	Inflammation markers (TNF-*α*, IL-1*β*)	Model control, *n* = 10 Curcumin, *n* = 10
Zou 2018	APOE^−/−^	9 w	/	HFD	Chow	Sigma ≥98%	16 w	1000 mg/kg/d dietary	150 mg/kg	Aortic sinus stained with Oil Red O	Lipid markers (Tc, TG, HDL, LDL)	Model control, *n* = 10 Curcumin, *n* = 10

HFD: high-fat diet; NCD: normal chow diet; i.v.: intravenous injection; i.g.: oral gavage; HE: hematoxylin-eosin; ^∗^Standardized dosage as Sharma [17] has reported.

**Table 2:** Summary of the anti-inflammation and antilipidemic effect of curcumin.

Outcome	Included experiments	*N*	MD	*I* ^2^%	*P* value	Egger's test
*(1) Effect on plasma lipid level*						
Tc	12 [8–9, 17–18, 29–30, 32–33]	179	-1.005 (-1.885, -0.124)	76.20%	0.025	0.226
TG	12 [8–9, 17–18, 29–30, 32–33]	179	-0.045 (-0.088, -0.002)	0.20%	0.042	0.063
HDL	9 [17–18, 29–30, 32]	109	-0.087 (-0.235, 0.060)	9.20%	0.245	0.975
LDL-c	7 [18, 29–30, 32–33]	108	-0.523 (-0.896, -0.149)	89.10%	0.006	0.081
*(2) Effect on plasma inflammatory responses*						
TNF-*α*^#^	6 [18, 29–31]	88	-56.641 (-86.848, -26.433)	92.30%	<0.001	0.036^∗^
IL-6	8 [17–18, 29–30]	89	-10.687 (-23.705, 2.331)	90.40%	0.108	0.753
IL-1*β*	4 [17, 31]	41	-5.089 (-8.559, -1.619)	0.00%	0.004	0.894

Tc: total cholesterol; TG: triglyceride; HDL: high-density lipoprotein; LDL-c: low-density lipoprotein cholesterol; TNF-*α*: tumor necrosis factor-*α*; IL-6: interleukin-6; IL-1*β*: interleukin-1*β*; *N*: total number of animals in the study; ^#^Two pairwise experiments were omitted; ^∗^Publication bias existed. *P* < 0.05 represents the significant difference.

**Table 3:** Results of metaregression analysis.

		Coef.	95% CI	*t* value	*P* value	
Continuous variable						
Publication year		0.053	(-0.219, 0.113)	-0.69	0.501	
Study length		0.005	(-0.128, 0.137)	0.08	0.937	
Age of mice		0.290	(-0.792, 0.213)	-1.25	0.234	
Binary variable						
Source of curcumin		1.026	(-2.361, 0.310)	-1.67	0.120	
Gender of mice		0.519	(-1.154, 2.192)	0.68	0.512	
Staining methods		0.545	(-1.817, 1.009)	-0.62	0.545	
Multicategorical variable^∗^						
Assessing location	Arch	0.717	(-2.818, 1.384)	-0.76	0.465	0.7276
	Sinus	0.695	(-2.770, 1.379)	-0.75	0.472	
	Whole	0.513	(-1.449, 2.475)	0.58	0.573	
Route of curcumin	Chow	0.527	(-1.165, 2.220)	0.69	0.507	0.1776
	Intravenous injection	0.758	(-1.443, 2.959)	0.76	0.464	

Coef.: coefficient; CI: confidence interval; ^∗^Dummy variable was applied for metaregression analysis of multicategorical variable. The former *P* value in metaregression analysis of multicategorical variable represents the comparison of each variable with the control variable (thoracic aorta or oral gavage). The latter *P* value represents a total statistical difference of assessing location area or route of curcumin. *P* < 0.05 was considered to be of statistical difference.

## Abbreviations

SMD: Standard mean difference
MD: Mean difference
RR: Ratio of means (means_experimental_/means_control_)
CI: Confidence interval
Tc: Total cholesterol
TG: Triglyceride
LDL-c: Low-density lipoprotein cholesterol
HDL: High-density lipoprotein
TNF-*α*: Tumor necrosis factor-*α*
IL-1*β*: Interleukin-1*β*
IL-6: Interleukin-6
BW: Based on weight
M_*φ*_: Macrophage
VSMC: Vascular smooth muscle cell
EC: Endothelial cell.

## Supplementary Materials

Supplementary Materials Figure S1: dose-response between overall doses of curcumin and aortic atherosclerotic lesion area. Univariate metaregression analysis to reveal the linear relationship between SMD of aortic lesion area and standard dosage of curcumin. Figure S2: sensitivity analysis of curcumin on plasma lipid level. Sensitivity analysis of curcumin on serum Tc (A), TG (B), LDL-c (C), and HDL (D). Tc: total cholesterol; TG: triglyceride; LDL-c: low-density lipoprotein cholesterol. Figure S3: sensitivity analysis of curcumin on plasma inflammatory response level. Sensitivity analysis of curcumin on serum TNF-*α* before omitting Hasan et al. [17] (A) and after (B), IL-6 (C), and IL-1*β* (D). TNF-*α*: tumor necrosis factor-*α*; IL-6: interleukin-6; IL-1*β*: interleukin-1*β*.
