# Supplementary material for: Efficacy of Curcumin on Aortic Atherosclerosis: A Systematic Review and Meta-Analysis in Mouse Studies and Insights into Possible Mechanisms
Source: Oxid Med Cell Longev. 2020 Jan 7;2020:1520747. doi: 10.1155/2020/1520747 (PMC6973199; doi:10.1155/2020/1520747)
Supplement: Supplementary Materials — Figure S1: dose-response between overall doses of curcumin and aortic atherosclerotic lesion area. Univariate metaregression analysis to reveal the linear relationship between SMD of aortic lesion area and standard dosage of curcumin. Figure S2: sensitivity analysis of curcumin on plasma lipid level. Sensitivity analysis of curcumin on serum Tc (A), TG (B), LDL-c (C), and HDL (D). Tc: total cholesterol; TG: triglyceride; LDL-c: low-density lipoprotein cholesterol. Figure S3: sensitivity analysis of curcumin on plasma inflammatory response level. Sensitivity analysis of curcumin on serum TNF-α before omitting Hasan et al. [17] (A) and after (B), IL-6 (C), and IL-1β (D). TNF-α: tumor necrosis factor-α; IL-6: interleukin-6; IL-1β: interleukin-1β. [file 1520747.f1.pdf]

Supplementary Figures

Efficacy of curcumin on aortic atherosclerosis: Meta-analysis of mice studies and insights into molecular mechanism

Firstly, a univariate meta-regression analysis was performed to clarify the relationship between SMD of aortic lesion area and overall dosage of curcumin included in our analyses.

Figure S1

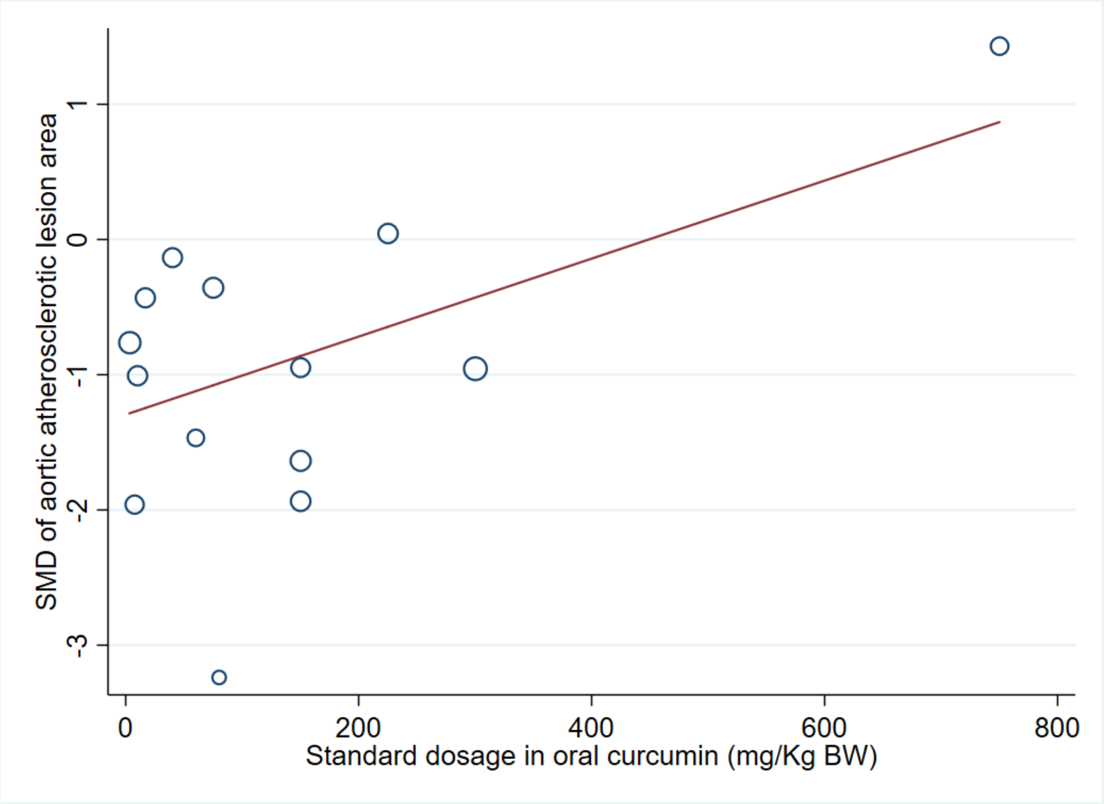

Figure S1. Dose-response between overall doses of curcumin and aortic atherosclerotic lesion area.

Univariate meta-regression analysis to reveal the linear relationship between SMD of aortic lesion area and standard dosage of curcumin.

Figure S2

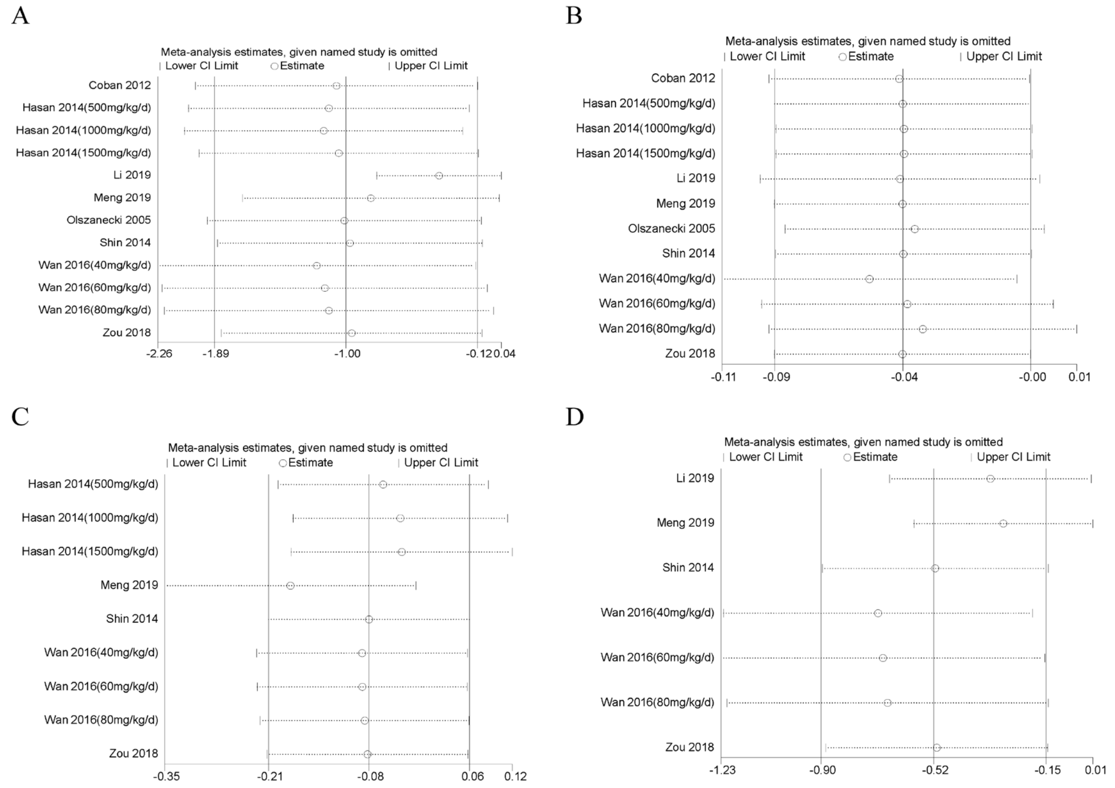

Figure S2. Sensitivity analysis of curcumin on plasma lipid level.

Sensitivity analysis of curcumin on serum Tc (A), TG (B), LDL-c (C) and HDL (D). Tc, total cholesterol; TG, triglyceride; LDL-c, low density lipoprotein cholesterol.

Figure S3

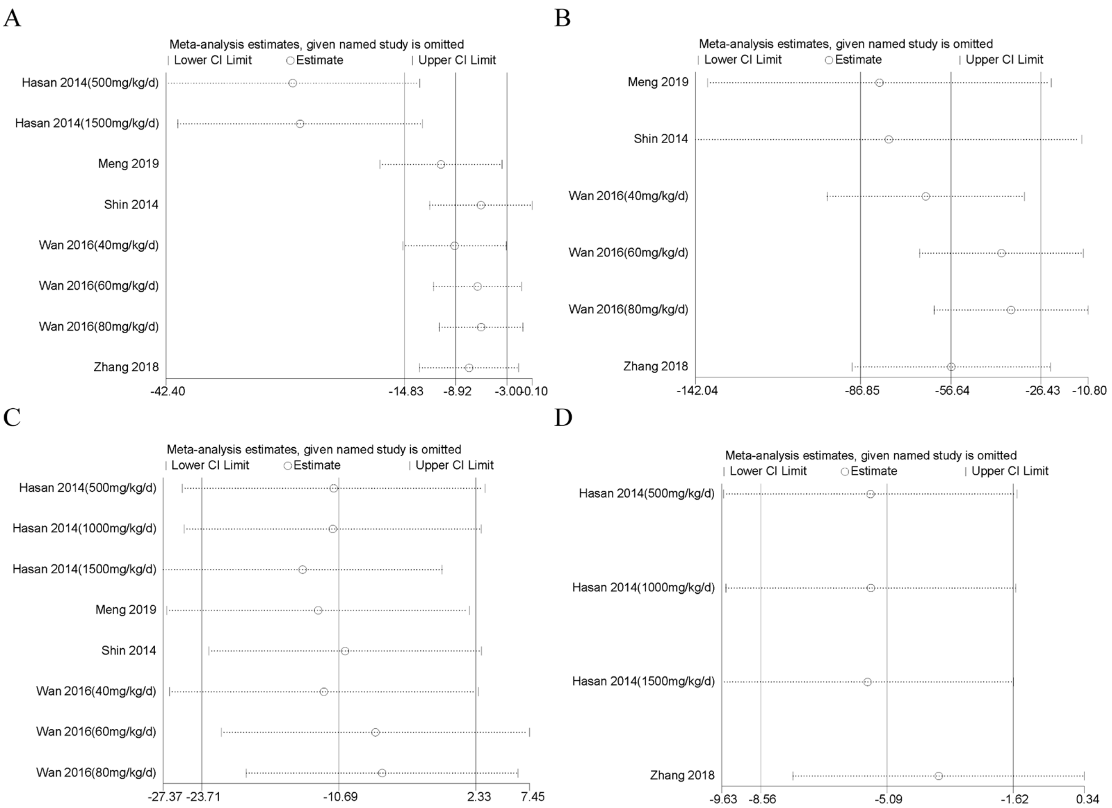

Figure S3. Sensitivity analysis of curcumin on plasma inflammatory response level

Sensitivity analysis of curcumin on serum TNF-α before omitting Hasan *et al.* (A) and after (B), IL-6 (C) and IL-1β (D). TNF-α, tumor necrosis factor α; IL-6, integrin 6; IL-1β, integrin 1β.
